# Evaluation of Serum Pentraxin-3 and Heavy Metal Levels in Patients with Acute Coronary Syndrome

**DOI:** 10.3390/toxics14100881

**Published:** 2026-09-30

**Authors:** Emre Gokcen, Hamza Enes Guclu, Vugar Ali Turksoy, Metin Ozsoy, Mikail Kusdogan, Vahit Demir, Levent Albayrak, Sevilay Vural, Mustafa Said Acar

**Affiliations:** 1 Department of Emergency Medicine, Faculty of Medicine, Yozgat Bozok University, 66100 Yozgat, Türkiyesevilayvural@yahoo.com (S.V.); 2 Department of Emergency Medicine, Erzurum City Hospital, 25240 Erzurum, Türkiye; 3 Department of Public Health, Faculty of Medicine, Yozgat Bozok University, 66100 Yozgat, Türkiye; 4 Department of Infectious Diseases and Clinical Microbiology, Ankara Training and Research Hospital, University of Health Sciences, 06230 Ankara, Türkiye; 5 Department of Cardiology, Aksaray Training and Research Hospital, 68200 Aksaray, Türkiye; dr.vdemir@hotmail.com; 6 Department of Emergency Medicine, Cide State Hospital, Cide, 37600 Kastamonu, Türkiye

**Keywords:** acute coronary syndrome, pentraxin-3, lead, heavy metals, vascular inflammation, biomarkers

## Abstract

Background: Vascular inflammation and vasculotoxic heavy metals have each been linked to acute coronary syndrome (ACS) but have rarely been studied together. This study compared serum pentraxin-3 (PTX-3), a proposed marker of local vascular inflammation, and whole-blood lead (Pb), arsenic (As), and nickel (Ni) between ACS patients and controls. Methods: In this single-center, cross-sectional case–control study, 50 patients with angiographically confirmed ACS were compared with 50 age- and sex-comparable emergency department controls with minor non-cardiac complaints. Results: PTX-3, Pb, As, and Ni were higher in ACS patients (all false discovery rate-adjusted q < 0.001). Between-group discrimination was highest for Pb (area under the curve [AUC] 0.962) and PTX-3 (AUC 0.951). PTX-3 and Pb remained independently associated with ACS after adjustment for hypertension, hyperlipidemia, and smoking and, separately, for age, diabetes, and renal function. Within the ACS group, PTX-3 was not significantly correlated with the Gensini score (ρ = 0.12, *p* = 0.41). Conclusions: PTX-3 and whole-blood Pb, As, and Ni were markedly elevated in ACS, with PTX-3 and Pb showing the strongest and most robust associations. Prospective multicenter studies in patients with suspected ACS are needed to confirm these findings and establish whether these biomarkers add value beyond established clinical assessment.

## 1. Introduction

Acute coronary syndromes (ACS) are a leading cause of cardiovascular mortality and a central focus of emergency department triage [1]. Although high-sensitivity cardiac troponins (hs-cTn) are the gold standard for detecting myocardial necrosis, they are insufficient for predicting the underlying vulnerable-plaque substrate and early atherothrombotic risk [1,2]. At the etiological level, five major modifiable risk factors—body mass index, systolic blood pressure, non-HDL cholesterol, current smoking, and diabetes—collectively account for just over half of incident cardiovascular disease globally [3]. Residual inflammatory risk also remains common among statin-treated patients despite intensive LDL-cholesterol lowering, highlighting the continued relevance of inflammatory pathways in atherosclerotic disease [4]. Because vascular inflammation governs every stage of the atherosclerotic plaque from its development through rupture and thrombosis, there is a need for next-generation biomarkers capable of reflecting local vascular injury and acute plaque instability at an early stage [2,4,5].

Among these markers, whereas the classical short pentraxin CRP is a systemic, late-rising acute-phase reactant produced in the liver under IL-6 stimulation, the long pentraxin pentraxin-3 (PTX-3) is synthesized locally and directly by endothelial cells, macrophages, and smooth muscle cells within the injured vessel wall [6]. This liver-independent production has led PTX-3 to be proposed as a marker of local inflammatory activation [6]. Circulating PTX-3 rises early during acute myocardial infarction and peaks earlier than CRP [7]. Prospective and meta-analytic data indicate that elevated PTX-3 levels in the acute phase are an independent prognostic marker for major adverse cardiovascular events and mortality [8,9].

In parallel, the emerging “environmental cardiology” perspective positions environmental exposures as an atherogenic dimension beyond traditional factors [10,11]. Chronic exposure to vasculotoxic metals such as lead (Pb), arsenic (As), and nickel (Ni) occurs through air, water, and food; these metals are increasingly recognized yet understudied cardiovascular risk factors. Experimental studies implicate oxidative stress, mitochondrial injury, endothelial dysfunction and NF-κB-related inflammation in their vascular toxicity, although mechanisms vary by metal and model [11,12,13,14]. Indeed, large population studies have even reported an independent association between environmental-level metal exposure and cardiovascular risk [15].

However, although PTX-3 and toxic-metal exposure have each been associated with cardiovascular risk separately, to our knowledge no study has examined these two components concurrently in an angiographically confirmed acute ischemic setting. Yet, given experimental evidence linking Pb, As, and Ni exposure to oxidative stress and inflammatory signaling [11,12,13,14], the possibility that these processes also contribute to PTX-3 up-regulation raises the prospect of a shared pathophysiological mechanism linking the two parameters.

On these grounds, we aimed to compare serum PTX-3 and whole-blood vasculotoxic-metal (Pb, As, Ni) concentrations between angiographically confirmed ACS patients and controls without known cardiovascular disease. Secondarily, we evaluated their ability to discriminate ACS cases from controls (ROC analysis), their associations with ACS after adjustment for selected conventional cardiovascular risk factors, and the relationship of PTX-3 with coronary disease severity (Gensini score). We hypothesized that serum PTX-3 and whole-blood metal concentrations would be elevated in ACS cases and associated with ACS independently of selected conventional cardiovascular risk factors.

## 2. Materials and Methods

### 2.1. Study Design and Setting

This single-center, cross-sectional case–control study was conducted at Yozgat Bozok University Faculty of Medicine, Yozgat, Türkiye, between February and August 2024. The study protocol was approved by the Yozgat Bozok University Clinical Research Ethics Committee (approval no. 2017-KAEK-189_2023.05.25_4), in accordance with the Declaration of Helsinki, and written informed consent was obtained from every participant.

### 2.2. Study Population

A total of 100 participants were enrolled: 50 patients with acute coronary syndrome (cases) and 50 controls of comparable age and sex. Cases were patients with ST-elevation myocardial infarction (STEMI) or non-ST-elevation myocardial infarction (NSTEMI) diagnosed according to the Fourth Universal Definition of Myocardial Infarction [16], all of whom underwent emergency coronary angiography. Controls were patients of similar age who presented to the emergency department outpatient clinic with minor, non-cardiac complaints, had no history of coronary artery disease, and in whom acute coronary syndrome was not clinically suspected. Exclusion criteria, applied to both groups, were age under 18 years; known coronary artery disease (previous myocardial infarction, percutaneous coronary intervention, or coronary artery bypass grafting); and conditions or treatments capable of altering inflammatory or heavy metal status, including active or chronic infection, chronic inflammatory or autoimmune disease, malignancy, moderate-to-severe renal or hepatic impairment, and systemic corticosteroid, immunosuppressive, or biologic therapy, as well as known occupational or environmental heavy metal exposure. Patients with non-ischemic conditions that may mimic acute coronary syndrome or elevate cardiac troponin—such as acute myocarditis, pericarditis, aortic dissection, or pulmonary embolism—and those with acute ischemic or hemorrhagic stroke, pregnancy, or recent major surgery or trauma were also excluded.

### 2.3. Clinical Assessment and Data Collection

Conventional cardiovascular risk factors (hypertension, hyperlipidemia, diabetes mellitus, and current smoking) were recorded at enrollment from documented medical history and self-reported smoking status. Serum creatinine was obtained from routine laboratory testing at enrollment, and eGFR was calculated using the race-free 2021 CKD-EPI creatinine equation [17].

### 2.4. Coronary Angiography and Assessment of Disease Severity

All ACS patients underwent emergency coronary angiography. The angiographic severity of coronary artery disease was quantified using the Gensini score [18], calculated by a single interventional cardiologist who was blinded to the biomarker results.

### 2.5. Blood Sampling and Biomarker Measurement

Peripheral venous blood samples were obtained from ACS patients at emergency department presentation, before coronary angiography and the administration of any related medication. Control samples were obtained at the emergency department outpatient clinic. Blood intended for PTX-3 determination was collected in biochemistry tubes and centrifuged at 3000 rpm for 10 min; the separated serum was aliquoted into polypropylene microtubes. Whole blood collected for metal analysis was retained without centrifugation. Serum and whole-blood aliquots were stored at −20 °C until analysis.

Serum pentraxin-3 (PTX-3) concentrations were determined using a commercially available human PTX-3 enzyme-linked immunosorbent assay (ELISA) kit in accordance with the manufacturer’s instructions at the Multidisciplinary Research Laboratory of Yozgat Bozok University Faculty of Medicine. Briefly, 50 µL of serum was transferred to the assay microplate, the kit reagents were added sequentially, and the colorimetric reaction was developed as specified by the manufacturer. Absorbance was measured at 450 nm using a microplate reader, and PTX-3 concentrations were quantified against the kit-supplied standard curve.

As part of routine emergency department evaluation, high-sensitivity cardiac troponin T (hs-cTnT) was measured by electrochemiluminescence immunoassay using Roche Diagnostics reagents (Basel, Switzerland). Values were retrieved from hospital records, and troponin was not measured in controls because ACS was not clinically suspected.

Whole-blood concentrations of tin (Sn), aluminum (Al), cobalt (Co), nickel (Ni), arsenic (As), cadmium (Cd), mercury (Hg), and lead (Pb) were quantified by inductively coupled plasma mass spectrometry (ICP-MS; iCAP Qc, Thermo Scientific, Waltham, MA, USA). Sample preparation was performed at the Multidisciplinary Research Laboratory of Yozgat Bozok University Faculty of Medicine, and instrumental analysis was conducted at the Yozgat Bozok University Science and Technology Application and Research Center. An acid-digestion procedure was adapted for whole blood. One-milliliter aliquots were transferred to 15 mL polypropylene tubes and digested with 5 mL of suprapure nitric acid (HNO_3_) and 2 mL of hydrogen peroxide (H_2_O_2_). Digestion was completed within 24 h, after which each digest was brought to a final volume of 10 mL with ultrapure water and stored at 4 °C until instrumental analysis. Quantification was based on an 11-point calibration curve, and analytical accuracy was verified using Seronorm WBL-2 certified reference material (Sero AS, Billingstad, Norway). Plasma power was set to 1500 W and nebulizer pressure to 2.98 bar, and the spray chamber temperature was measured to be 3.0 °C. High-purity argon gas was used as gas; plasma gas was set to 0.7 L/min, and nebulizer gas was set to 0.9 L/min. The measurement was performed in 0.01 s. Measurements of samples and standard samples were repeated a total of 3 times. The results were obtained as the mean of 3 readings, and it was determined that the relative standard deviation (RSD) did not surpass 5%. Measurement of the blank was performed using 3% nitric acid solution. The *r*^2^ values of the calibration curves were determined to be at least 0.9992.

### 2.6. Statistical Analysis

Statistical analyses were performed in IBM SPSS Statistics (v24) and jamovi (v2.7.38, based on R v4.5.0). Normality was assessed with the Shapiro–Wilk test. Baseline characteristics were compared in SPSS using Student’s independent-samples *t*-test (age; mean ± SD), the Mann–Whitney U test (body mass index, serum creatinine and eGFR; median [IQR]), and the Pearson chi-square or Fisher’s exact test (categorical variables; counts [%]). Biomarker concentrations were compared with the Mann–Whitney U test, with Benjamini–Hochberg false discovery rate correction across the biomarker panel [19]. Discriminative performance was assessed by ROC analysis, reporting the area under the curve (AUC) with its 95% confidence interval (CI) and Youden-based cut-offs regarded as exploratory; because cut-offs were derived and evaluated in the same sample, bootstrap internal validation (1000 replications) was performed to obtain optimism-corrected estimates. Because several biomarkers produced near-complete separation, under which standard maximum-likelihood estimation is unstable, associations with ACS were modeled by Firth penalized logistic regression with profile penalized-likelihood confidence intervals [20,21]. Markers significant in univariable analysis were entered into a parsimonious model retaining the two most discriminative markers, and in a sensitivity analysis this model was additionally adjusted for hypertension, hyperlipidemia, and smoking. A second sensitivity model adjusted PTX-3 and Pb for age, diabetes mellitus, and eGFR, potential determinants of metal concentrations and inflammatory biomarkers. It was evaluated separately from the hypertension/hyperlipidemia/smoking model to limit overparameterization given only 50 ACS events; both sensitivity models were considered exploratory. Within the ACS group, Spearman correlations assessed the relationships of PTX-3, lead, arsenic and nickel with the Gensini score, PTX-3 with the metals, and hs-cTnT with these biomarkers and the Gensini score. Comparisons between STEMI and NSTEMI subgroups were performed with the Mann–Whitney U test. The STEMI–NSTEMI subgroup comparisons and the hs-cTnT correlation analyses were post hoc and exploratory. Subgroup comparisons and all correlation analyses are reported without correction for multiple comparisons. Pooled inter-biomarker correlations were not reported, as they are confounded by group membership. A two-sided *p* (or q) < 0.05 was considered significant.

## 3. Results

### 3.1. Baseline Characteristics of the Study Population

A total of 100 participants were enrolled, comprising 50 patients with acute coronary syndrome (ACS) who underwent emergency coronary angiography and 50 control participants of comparable age and sex. The two groups did not differ significantly in age (64.3 ± 12.6 vs. 63.9 ± 12.6 years; *p* = 0.868), the proportion of male participants (64% in both groups; *p* = 1.000), or body mass index (BMI; 27.0 [25.4–28.1] vs. 26.8 [26.1–28.2] kg/m^2^; *p* = 0.764). Conventional cardiovascular risk factors were more frequent among ACS patients: hypertension (38% vs. 12%; *p* = 0.003), hyperlipidemia (40% vs. 18%; *p* = 0.015), and current smoking (46% vs. 24%; *p* = 0.021) all differed significantly, whereas diabetes mellitus (18% vs. 8%; *p* = 0.137) and chronic obstructive pulmonary disease (6% vs. 6%; *p* = 1.000) did not. Serum creatinine and estimated glomerular filtration rate (eGFR) did not differ between groups (creatinine 0.79 [0.65–0.88] vs. 0.79 [0.56–0.96] mg/dL, *p* = 0.544; eGFR 94.0 [85.4–103.0] vs. 96.4 [75.2–109.5] mL/min/1.73 m^2^, *p* = 0.634). Among ACS patients, the median Gensini score was 68.0 (interquartile range [IQR], 35.8–98.1; range, 0–172). Baseline characteristics are summarized in Table 1.

### 3.2. Blood Heavy Metals and Pentraxin-3 Concentrations

**Figure 1:**
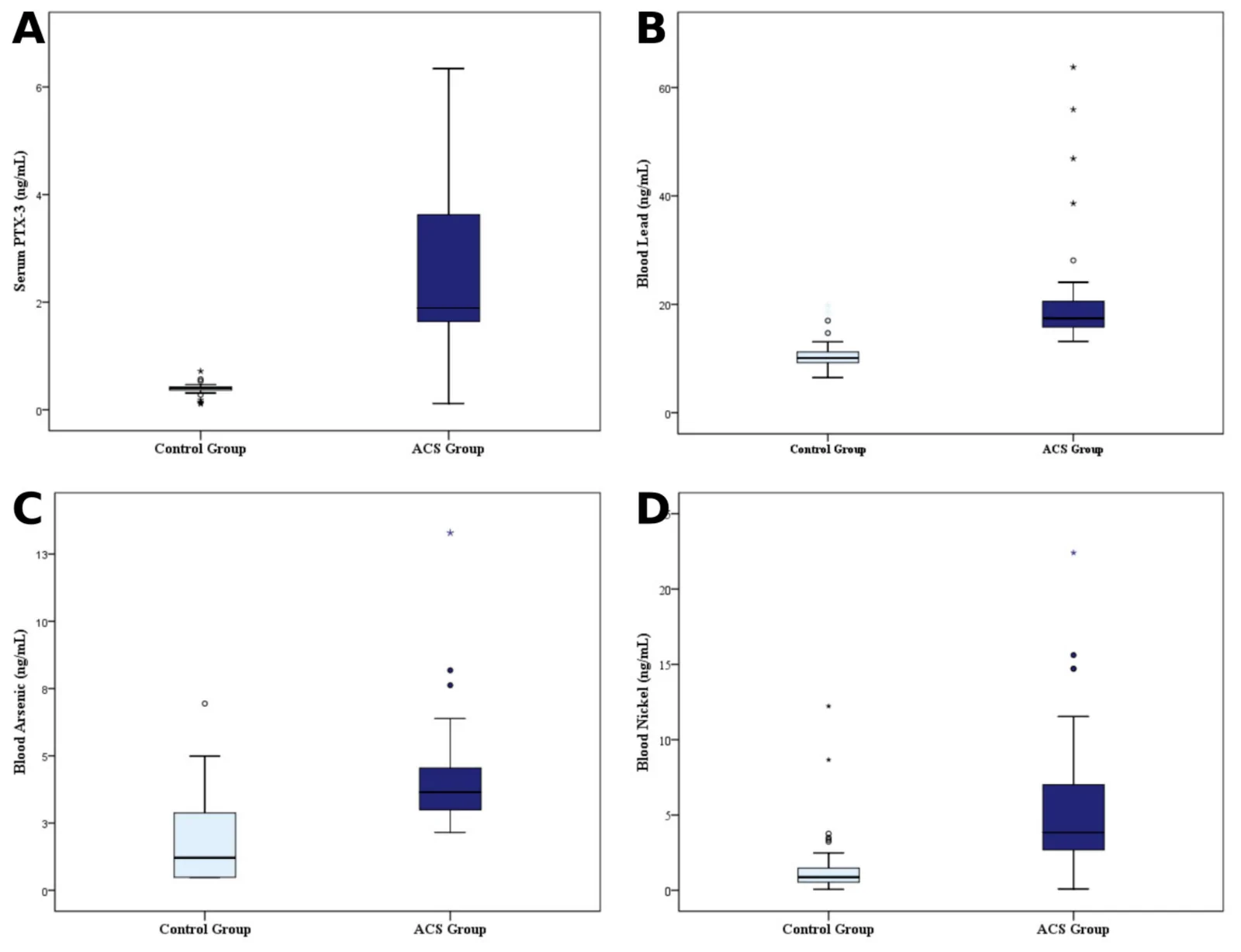
Serum pentraxin-3 (**A**) and whole-blood lead (**B**), arsenic (**C**), and nickel (**D**) concentrations in patients with acute coronary syndrome (ACS) versus controls.

Because all biomarker distributions departed from normality (Shapiro–Wilk *p* < 0.05), between-group comparisons were performed with the Mann–Whitney U test, and the Benjamini–Hochberg false discovery rate (FDR) procedure was applied to account for multiple comparisons; both raw *p*-values and FDR-adjusted q-values are reported (Table 2). Serum pentraxin-3 (PTX-3) was markedly higher in ACS patients than in controls (1.89 [1.64–3.51] vs. 0.40 [0.37–0.42] ng/mL; *p* < 0.001, q < 0.001). Among heavy metals, lead (Pb), arsenic (As), and nickel (Ni) were substantially higher in ACS patients, each with q < 0.001, whereas tin (Sn) and aluminum (Al) were modestly elevated (q = 0.016 and 0.040, respectively). In contrast, cobalt (Co) and mercury (Hg) were lower in ACS patients (q = 0.040 for both), and cadmium (Cd) did not differ between groups (q = 0.828). The distributions of the four biomarkers most strongly associated with ACS—PTX-3, lead, arsenic, and nickel—are shown in Figure 1.

### 3.3. Between-Group Discrimination

**Figure 2:**
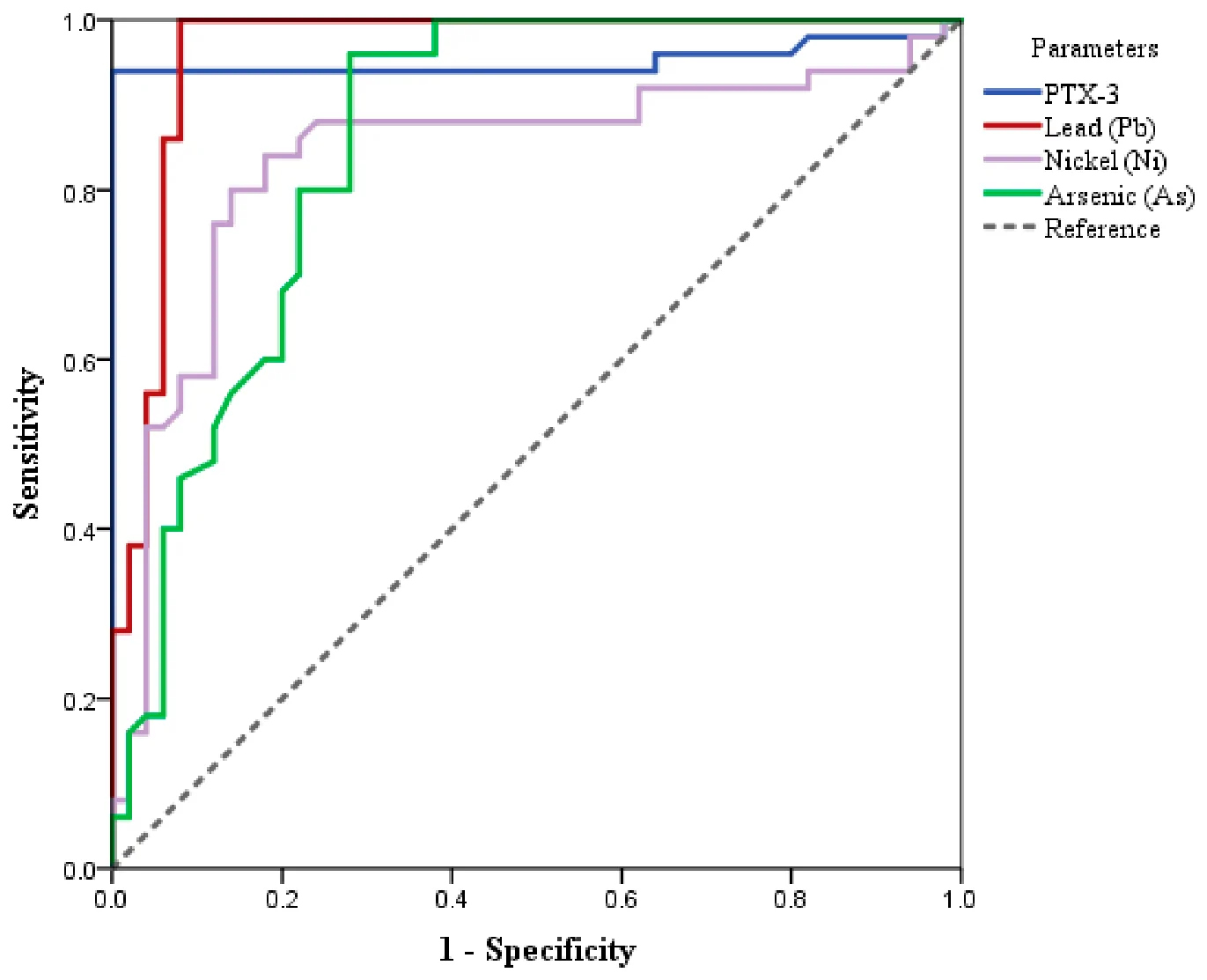
Receiver operating characteristic (ROC) curves of serum pentraxin-3 and whole-blood lead, nickel, and arsenic for discrimination of acute coronary syndrome from controls. The diagonal reference line denotes no discrimination (AUC = 0.5).

The ability of the four biomarkers most strongly associated with ACS to discriminate between the groups was evaluated by receiver operating characteristic (ROC) analysis (Table 3; Figure 2). Lead and PTX-3 discriminated cases from controls most strongly (area under the curve [AUC] 0.962 and 0.951, respectively), followed by arsenic and nickel (AUC 0.859 and 0.838). Because candidate cut-off values were derived and evaluated in the same sample, they should be regarded as exploratory; internal validation by bootstrap resampling (1000 replications) indicated negligible optimism, with optimism-corrected AUCs essentially unchanged from the apparent values.

### 3.4. Independent Associations of Biomarkers with Acute Coronary Syndrome

Because PTX-3 and Pb almost completely separated the two groups, yielding unstable maximum-likelihood estimates, associations with ACS were modeled using Firth penalized logistic regression, with 95% CIs obtained from the profile penalized likelihood (Table 4). In univariable analysis, all four candidate biomarkers were significantly associated with ACS. A parsimonious multivariable model incorporating the two most discriminative markers retained both PTX-3 (adjusted odds ratio [aOR] 38.0, 95% CI 6.6–1261.4; *p* < 0.001) and Pb (aOR 1.66 per 1 ng/mL, 95% CI 1.25–2.67; *p* < 0.001), each of which remained independently associated with ACS; a model additionally including Ni and As was unstable owing to collinearity and separation and was not adopted.

In a sensitivity analysis additionally adjusting for hypertension, hyperlipidemia, and smoking, both PTX-3 and Pb remained independently associated with ACS (both *p* < 0.01). In a second sensitivity analysis adjusting for age, diabetes mellitus, and eGFR, PTX-3 (aOR 41.3, 95% CI 6.3–4153.2; *p* < 0.001) and Pb (aOR 1.55 per 1 ng/mL, 95% CI 1.19–2.53; *p* = 0.001) remained independently associated with ACS; point estimates changed by <10% from the primary two-marker model (Table 5). Age, diabetes mellitus, and eGFR were not independently associated with ACS; the diabetes estimate was imprecise because of sparse observations.

### 3.5. Correlations Within the ACS Group

Within the ACS group, PTX-3 concentration was not significantly correlated with angiographic disease severity as measured by the Gensini score (Spearman ρ = 0.12, *p* = 0.41). No significant correlations with the Gensini score were observed for Pb, Ni, or As.

PTX-3 was not significantly correlated with lead (ρ = −0.04, *p* = 0.77) or arsenic (ρ = 0.12, *p* = 0.41), whereas a weak inverse correlation was observed with nickel (ρ = −0.29, *p* = 0.038).

### 3.6. Infarction Subtype, Cardiac Troponin, and Biomarker Levels Within the ACS Group

Among 50 ACS patients, 24 (48%) had STEMI and 26 (52%) NSTEMI. Median hs-cTnT was 1370.2 [309.4–5792.4] ng/L and was higher in STEMI than NSTEMI (*p* = 0.042), as was the Gensini score (*p* < 0.001). PTX-3 did not differ by subtype (1.95 [1.69–3.72] vs. 1.83 [1.44–2.93] ng/mL, *p* = 0.641), nor did lead, arsenic, or nickel (all *p* > 0.30; Table 6).

Within ACS, hs-cTnT was not correlated with PTX-3 (ρ = 0.03, *p* = 0.83) or with lead, arsenic, or nickel (ρ = −0.23 to 0.20, all *p* > 0.10), but correlated modestly with the Gensini score (ρ = 0.31, *p* = 0.026).

## 4. Discussion

One of the principal findings of the present study was that serum PTX-3 concentrations were markedly elevated in patients with acute coronary syndrome (ACS) and showed strong discrimination between ACS cases and controls. The association of PTX-3 with ACS persisted in both sensitivity models adjusting for selected cardiovascular risk factors and potential biomarker-related confounders. This independent and strong association is consistent with the biological profile and kinetic behavior of PTX-3 described in the literature. As reported, in contrast to C-reactive protein (CRP) the prototypical short pentraxin, produced systemically in the liver under interleukin-6 (IL-6) stimulation, PTX-3 is synthesized locally at the site of inflammation and tissue injury by endothelial cells, macrophages, and smooth muscle cells within the vessel wall [22]. Owing to this local production profile, circulating PTX-3 may reflect local vascular and myocardial involvement; during acute myocardial infarction it rises rapidly and peaks earlier than CRP [7]. Consistent with this, a meta-analysis demonstrated that elevated PTX-3 levels in patients with ACS are a strong and independent predictor of cardiovascular mortality and adverse cardiac events [8].

A further finding was the absence of a significant correlation between PTX-3 and the Gensini score. The Gensini score quantifies angiographic stenosis severity but does not directly assess vascular inflammation, plaque composition, or instability. Previous studies have also yielded inconsistent findings regarding anatomical coronary disease burden: PTX-3 was not associated with the presence of CAD or the number of significantly stenosed vessels on coronary CT angiography [23], whereas one small study in patients with stable angina reported a modest positive correlation with the Gensini score [24]. Taken together with previous studies, the present finding suggests that a consistent relationship between PTX-3 and anatomical coronary disease burden has not been established; however, the relatively small correlation sample does not exclude a weak or moderate association. Separately, imaging studies using optical coherence tomography, intravascular ultrasound, and computed tomography have associated PTX-3 with plaque-vulnerability features, including thin-cap fibroatheroma [25,26,27], plaque rupture [25,27], and lower CT plaque density [24], as well as with impaired post-PCI myocardial perfusion [27]. From a prognostic perspective, plasma PTX-3 levels were reported not to predict recurrent coronary events after myocardial infarction [28]. These observations, together with broader evidence linking PTX-3 to vascular inflammation and atherosclerotic biology [29,30], suggest that PTX-3 may be associated with aspects of coronary pathophysiology beyond angiographic stenosis severity; however, the present study did not directly assess vascular inflammation or plaque morphology.

PTX-3 did not differ between STEMI and NSTEMI and was not correlated with hs-cTnT; hs-cTnT was higher in STEMI and correlated modestly with the Gensini score. These post hoc analyses were exploratory and uncorrected for multiple comparisons. Because hs-cTnT was measured only in ACS patients, the study could not directly compare PTX-3 and hs-cTnT diagnostic performance or assess the incremental value of PTX-3. Prospective studies measuring both markers in all participants are required.

Exposure to heavy metals—particularly lead, arsenic, and environmental toxic elements—has been linked to cardiovascular disease risk and the atherosclerotic process in experimental and epidemiological studies through the triggering of vascular injury at the cellular level [11,31]. Among the heavy metals analyzed, lead (Pb), arsenic (As), and nickel (Ni) were markedly higher in the ACS group than in controls (Pb: 17.43 vs. 10.08 ng/mL; As: 3.65 vs. 1.21 ng/mL; Ni: 3.85 vs. 0.87 ng/mL; q < 0.001 for all), and all three elements exhibited substantial between-group discrimination (AUC 0.962, 0.859, and 0.838, respectively), supporting an association of these elements with ACS.

In this context, lead, a principal vasculotoxic agent, is closely linked to the pathogenesis of hypertension and the development of atherosclerosis [11]. In prospective NHANES cohorts, lead has been associated with long-term cardiovascular and ischemic mortality risk even at low blood concentrations [15,32]. The present cross-sectional findings complement rather than confirm these observations: in this cohort, whole-blood lead had the highest observed AUC for discrimination between ACS cases and controls (AUC = 0.962; sensitivity 100%, specificity 92%) and was associated with ACS in the primary two-marker model (aOR = 1.66; *p* < 0.001), with the association also persisting in both sensitivity models. Similarly, arsenic exposure has been associated with the risk of ischemic heart disease and myocardial infarction in prospective epidemiological studies [11,33], and clinical case–control studies have reported significant correlations with coronary artery occlusion and acute myocardial infarction [34]. Whole-blood arsenic concentrations were likewise approximately three-fold higher in ACS cases than in controls and showed substantial between-group discrimination (AUC = 0.859; univariable OR = 2.87), consistent with these epidemiological and clinical data.

Nickel, by contrast, has been examined in the cardiovascular literature largely through indirect data such as subclinical atherosclerosis, urinary excretion, or metabolic abnormalities rather than in relation to acute events [35,36,37], yet a marked between-group difference was observed in the present cohort. Whole-blood nickel concentrations were approximately 4.4-fold higher in ACS patients (q < 0.001), showed a significant association with ACS in univariable analysis (OR = 1.69; *p* < 0.001), and exhibited substantial between-group discrimination (AUC = 0.838; sensitivity 80%, specificity 86%). Given the limited evidence relating nickel to acute cardiovascular events, the marked between-group difference observed in the present cohort warrants further investigation of nickel in the acute ischemic setting.

The concurrent elevation of whole-blood vasculotoxic metals, particularly Pb, As, and Ni, and PTX-3 in the present ACS cohort may suggest the presence of shared biological pathways. Experimental evidence may provide a plausible framework for this association: lead exposure has been reported to increase reactive oxygen species (ROS), impair antioxidant defenses, and promote lipid peroxidation [12]. Similarly, arsenic has been reported to induce cellular redox imbalance through ROS accumulation [38], while nickel compounds can provoke oxidative stress in vascular and epithelial cells [39,40]. In experimental systems, Pb, As, and Ni have also been reported to activate nuclear factor kappa B (NF-κB) signaling and increase the expression of pro-inflammatory cytokines, including TNF-α and IL-1β [12,13,41,42,43].

Pro-inflammatory cytokines, particularly IL-1β and TNF-α, may stimulate PTX-3 expression in vascular and inflammatory cells [22,44]. Molecular studies in palmitic acid-treated human umbilical vein endothelial cell (HUVEC) models have further linked PTX-3 induction to the IKK/IκB/NF-κB axis and suggested that PTX-3 overexpression may contribute to endothelial injury and dysfunction [45,46]. Together, these experimental findings may provide a biologically plausible framework linking metal-related oxidative stress and vascular inflammatory signaling. However, the concurrent elevations observed in the present cohort do not demonstrate such a pathway directly; this interpretation remains hypothesis-generating.

As secondary, exploratory findings, cadmium levels did not differ between groups, whereas cobalt and mercury were marginally lower in the ACS group (q = 0.040 for both). The absence of a between-group difference for cadmium may relate to the fact that cadmium accumulates primarily in the renal cortex and that urinary cadmium is the biomarker that best reflects cumulative body burden; a single whole-blood concentration may therefore not fully capture chronic tissue accumulation or cardiovascular risk [47,48]. The lower cobalt and mercury levels in the ACS group should be interpreted with methodological caution rather than as evidence of a direct biological protective effect; the borderline significance of both elements after multiple-testing correction and their low baseline concentrations suggest that these differences may reflect analytical variability or unmeasured dietary factors.

In the present study, levels of the vasculotoxic metals Pb, As, and Ni and of PTX-3 were markedly higher in patients with ACS than in controls, with PTX-3 (AUC 0.951) and Pb (AUC 0.962) showing particularly strong between-group discrimination. In addition, the associations of PTX-3 and Pb with ACS were maintained across sensitivity analyses accounting for different cardiovascular risk factors and potential confounders. Previous studies have shown that PTX-3 is produced by vascular endothelial cells and macrophages in response to local inflammatory stimuli and rises early during acute myocardial infarction [6,7,8], while Pb, As, and Ni have been shown to promote vascular oxidative and inflammatory injury [31,49]. Environmental metal exposure has also been proposed as a contributor to cardiovascular risk [49,50]. Taken together with this literature, the present findings may point to a possible “environmental exposure–vascular inflammation” link beyond selected conventional cardiovascular risk factors. However, the PTX-3 (≥1.26 ng/mL) and Pb (≥13.16 ng/mL) cut-offs, derived and evaluated in the same sample, are not clinically validated diagnostic thresholds and therefore require validation in independent prospective cohorts reflecting routine clinical practice.

This study has several limitations. Controls presented with minor non-cardiac complaints rather than suspected ACS, which may have increased between-group differences, particularly for PTX-3, and limits generalizability to chest-pain populations. Angiography was performed only in ACS patients, and biomarker cut-offs were derived and evaluated in the same cohort; prospective external validation is therefore required. The single-center design and 50 ACS events limited statistical power and multivariable model complexity. STEMI/NSTEMI subgroup analyses were also underpowered, and the absence of a PTX-3 difference should not be interpreted as equivalence. Residual confounding remains possible because medication use and important determinants of metal exposure, including diet, drinking water, occupation, and residence, were not systematically collected. Wide PTX-3 confidence intervals further limit precision, and the multivariable analyses should therefore be considered exploratory. The cross-sectional case–control design also precludes causal inference.

C-reactive protein and white blood cell count were not standardized across groups, precluding assessment of the incremental value of PTX-3 over routine inflammatory markers. Symptom-to-sampling time was not systematically documented and may have affected PTX-3 concentrations. Whole-blood metals were measured once, and their exposure windows differ; these measurements therefore primarily reflect circulating concentrations at presentation rather than cumulative exposure and cannot establish that long-term metal exposure preceded or causally contributed to ACS.

## 5. Conclusions

In this case–control study, PTX-3 and the vasculotoxic metals Pb, As, and Ni were markedly elevated in patients with ACS and showed strong between-group discrimination. PTX-3 and Pb remained independently associated with ACS after adjustment for selected cardiovascular risk factors and potential confounders.

The absence of a significant association between PTX-3 and angiographic disease severity suggests that PTX-3 may reflect a pathophysiological dimension not captured by anatomical stenosis burden. In contrast, the lack of a consistent positive relationship between PTX-3 and the measured metals within the ACS group indicates that their concurrent elevation should not be interpreted as evidence of a direct metal–PTX-3 biological pathway.

These findings suggest that PTX-3 and circulating metal concentrations may be associated with different pathophysiological aspects of ACS. Prospective multicenter studies in clinically representative patients presenting with suspected ACS are needed to establish the external validity of these findings and determine whether these biomarkers provide incremental value beyond established clinical assessment.

## Figures and Tables

**Table 1:** Baseline demographic and clinical characteristics of the study groups.

Characteristic	ACS (*n* = 50)	Control (*n* = 50)	*p*
Age, years, mean ± SD	64.3 ± 12.6	63.9 ± 12.6	0.868 †
Male sex, *n* (%)	32 (64)	32 (64)	1.000 §
BMI, kg/m^2^, median [IQR]	27.0 [25.4–28.1]	26.8 [26.1–28.2]	0.764 ‡
Serum creatinine, mg/dL, median [IQR]	0.79 [0.65–0.88]	0.79 [0.56–0.96]	0.544 ‡
eGFR [17], mL/min/1.73 m^2^, median [IQR]	94.0 [85.4–103.0]	96.4 [75.2–109.5]	0.634 ‡
Hypertension, *n* (%)	19 (38)	6 (12)	0.003 §
Hyperlipidemia, *n* (%)	20 (40)	9 (18)	0.015 §
Current smoking, *n* (%)	23 (46)	12 (24)	0.021 §
Diabetes mellitus, *n* (%)	9 (18)	4 (8)	0.137 §
COPD, *n* (%)	3 (6)	3 (6)	1.000 ‖

† Student’s *t*-test; ‡ Mann–Whitney U test; § Pearson χ^2^ test; ‖ Fisher’s exact test. BMI, body mass index; COPD, chronic obstructive pulmonary disease; IQR, interquartile range; SD, standard deviation; eGFR, estimated glomerular filtration rate.

**Table 2:** Biomarker concentrations in ACS patients versus controls.

Biomarker (ng/mL)	ACS (*n* = 50)	Control (*n* = 50)	Dir.	*p*	q (FDR)
PTX-3	1.89 [1.64–3.51]	0.40 [0.37–0.42]	↑	<0.001	<0.001
Lead (Pb)	17.43 [15.86–20.39]	10.08 [9.23–11.16]	↑	<0.001	<0.001
Arsenic (As)	3.65 [3.00–4.51]	1.21 [0.48–2.86]	↑	<0.001	<0.001
Nickel (Ni)	3.85 [2.69–6.90]	0.87 [0.54–1.44]	↑	<0.001	<0.001
Tin (Sn)	1.07 [0.51–1.90]	0.51 [0.37–1.24]	↑	0.009	0.016
Aluminum (Al)	1.79 [1.26–2.39]	1.50 [0.25–2.29]	↑	0.035	0.040
Cobalt (Co)	0.11 [0.05–0.14]	0.14 [0.08–0.23]	↓	0.028	0.040
Mercury (Hg)	0.04 [0.04–0.07]	0.06 [0.04–0.11]	↓	0.036	0.040
Cadmium (Cd)	1.85 [1.47–2.30]	2.19 [1.13–2.77]	—	0.828	0.828

Values are median [IQR]. *p*, Mann–Whitney U test; q, Benjamini–Hochberg FDR-adjusted *p*-value. Dir., direction in ACS relative to controls (↑, higher in ACS; ↓, lower in ACS; —, no significant difference). FDR, false discovery rate; IQR, interquartile range; PTX-3, pentraxin-3.

**Table 3:** Between-group discrimination of biomarkers for ACS.

Biomarker	AUC (95% CI)	Optimism-Corr. AUC	Cut-Off (ng/mL)	Se (%)	Sp (%)
Lead (Pb)	0.962 (0.923–1.000)	0.962	≥13.16	100.0	92.0
PTX-3	0.951 (0.907–0.995)	0.951	≥1.26	94.0	100.0
Arsenic (As)	0.859 (0.784–0.933)	0.859	≥2.43	96.0	72.0
Nickel (Ni)	0.838 (0.758–0.917)	0.839	≥2.26	80.0	86.0

AUC, area under the ROC curve; CI, confidence interval; Se, sensitivity; Sp, specificity. Cut-off values were determined by the Youden index and are exploratory. Optimism-corrected AUCs were obtained by bootstrap internal validation (1000 replications).

**Table 4:** Firth penalized logistic regression for association with ACS.

Biomarker	Univariable OR (95% CI)	*p*	Multivariable aOR (95% CI)	*p*
PTX-3	163.7 (28.2–3971.1)	<0.001	38.0 (6.6–1261.4)	<0.001
Lead (Pb)	2.12 (1.66–2.94)	<0.001	1.66 (1.25–2.67)	<0.001
Nickel (Ni)	1.69 (1.35–2.24)	<0.001	Not retained *	—
Arsenic (As)	2.87 (1.96–4.62)	<0.001	Not retained *	—

Odds ratios are expressed per 1 ng/mL increase. * Excluded from the parsimonious multivariable model owing to collinearity and separation. aOR, adjusted odds ratio; CI, confidence interval; OR, odds ratio.

**Table 5:** Sensitivity analysis additionally adjusted for age, diabetes mellitus, and eGFR.

Variable	aOR (95% CI)	*p*
PTX-3, ng/mL	41.3 (6.3–4153.2)	<0.001
Lead (Pb), ng/mL	1.55 (1.19–2.53)	0.001
Age, years	1.05 (0.91–1.23)	0.509
Diabetes mellitus	6.71 (0.31–232.7) †	0.207
eGFR, mL/min/1.73 m^2^	1.02 (0.94–1.11)	0.595

Odds ratios are per 1 ng/mL increase in PTX-3 and Pb, 1 year in age, and 1 mL/min/1.73 m^2^ in eGFR; diabetes mellitus is compared with its absence. † The diabetes estimate was imprecise because of sparse observations. aOR, adjusted odds ratio; CI, confidence interval; eGFR, estimated glomerular filtration rate.

**Table 6:** Clinical characteristics of the ACS group by infarction subtype (*n* = 50).

Characteristic	STEMI (*n* = 24)	NSTEMI (*n* = 26)	*p*
hs-cTnT, ng/L	3620.4 [672.3–9692.0]	604.8 [240.2–2847.0]	0.042
Gensini score	102.2 [83.0–124.8]	42.2 [20.6–51.8]	<0.001
PTX-3, ng/mL	1.95 [1.69–3.72]	1.83 [1.44–2.93]	0.641
Lead (Pb), ng/mL	17.03 [15.95–19.24]	17.86 [15.51–21.11]	0.846
Arsenic (As), ng/mL	3.51 [2.91–4.40]	3.85 [3.19–4.51]	0.322
Nickel (Ni), ng/mL	3.64 [2.71–6.73]	4.11 [2.45–6.90]	0.823

Values are median [IQR]. *p*-values were calculated using the Mann–Whitney U test; subgroup comparisons are exploratory. Six hs-cTnT values exceeded 10,000 ng/L and were analyzed at that limit. hs-cTnT, high-sensitivity cardiac troponin T; IQR, interquartile range; NSTEMI, non-ST-elevation myocardial infarction; PTX-3, pentraxin-3; STEMI, ST-elevation myocardial infarction.

## Data Availability

The data presented in this study are available from the corresponding author upon reasonable request. As the data consist of identifiable patient-level clinical and laboratory records, any data sharing is subject to prior approval by the institutional ethics committee of Yozgat Bozok University and the execution of a data-sharing agreement, in accordance with patient privacy and ethical restrictions.
